# Central Nervous System Pathology Progresses Independently of KC and CXCR2 in Globoid-Cell Leukodystrophy

**DOI:** 10.1371/journal.pone.0064647

**Published:** 2013-06-03

**Authors:** Adarsh S. Reddy, Jigisha R. Patel, Carole Vogler, Robyn S. Klein, Mark S. Sands

**Affiliations:** 1 Department of Internal Medicine, Washington University School of Medicine, St. Louis, Missouri, United States of America; 2 Department of Pathology and Immunology, Washington University School of Medicine, St. Louis, Missouri, United States of America; 3 Department of Anatomy and Neurobiology, Washington University School of Medicine, St. Louis, Missouri, United States of America; 4 Department of Pathology, St. Louis University School of Medicine, St. Louis, Missouri, United States of America; University of South Florida, United States of America

## Abstract

Globoid-cell Leukodystrophy (GLD; Krabbe’s disease) is a rapidly progressing inherited demyelinating disease caused by a deficiency of the lysosomal enzyme Galactosylceramidase (GALC). Deficiency of GALC leads to altered catabolism of galactosylceramide and the cytotoxic lipid, galactosylsphingosine (psychosine). This leads to a rapidly progressive fatal disease with spasticity, cognitive disability and seizures. The murine model of GLD (Twitcher; GALC−/−) lacks the same enzyme and has similar clinical features. The deficiency of GALC leads to oligodendrocyte death, profound neuroinflammation, and the influx of activated macrophages into the CNS. We showed previously that keratinocyte chemoattractant factor (KC) is highly elevated in the CNS of untreated Twitcher mice and significantly decreases after receiving a relatively effective therapy (bone marrow transplantation combined with gene therapy). The action of KC is mediated through the CXCR2 receptor and is a potent chemoattractant for macrophages and microglia. KC is also involved in oligodendrocyte migration and proliferation. Based on the commonalities between the disease presentation and the functions of KC, we hypothesized that KC and/or CXCR2 contribute to the pathogenesis of GLD. Interestingly, the course of the disease is not significantly altered in KC- or CXCR2-deficient Twitcher mice. There is also no alteration in inflammation or demyelination patterns in these mice. Furthermore, transplantation of CXCR2-deficient bone marrow does not alter the progression of the disease as it does in other models of demyelination. This study highlights the role of multiple redundant cytokines and growth factors in the pathogenesis of GLD.

## Introduction

Globoid-cell leukodystrophy (GLD, Krabbe’s disease) is a rapidly progressive demyelinating disease with an autosomal recessive inheritance [1]. The disease is caused by a deficiency of the lysosomal enzyme galactosylceramidase (GALC). In the absence of GALC activity, the cytotoxic sphingolipid, galactosylsphingosine (psychosine) accumulates in the central (CNS) and peripheral nervous systems (PNS) [2]. Oligodendrocytes are particularly susceptible to elevated levels of psychosine [3]–[5]. Oligodendrocyte dysfunction and subsequent death are prominent features of GLD [3], [6]. The murine model of GLD (the Twitcher mouse) is deficient in GALC activity and shares many of the biochemical and histological features of the human disease [7]. Hence, the Twitcher mouse has been widely used to better understand the underlying pathogenesis and develop effective therapies for GLD.

CNS inflammation is a prominent histopathologic feature of GLD and is characterized by the presence of globoid cells (macrophages with engulfed myelin debris) and activated astrocytes in the CNS [8]–[10]. There is also an increase in pro-inflammatory cytokines and chemokines as well as an increase of T-cells and B-cells in the CNS of these animals [11]–[13]. It appears that inflammation could be a disease-altering target when considering treatment for GLD. Deletion of MHC Ia has been shown to alter the course of the disease in the Twitcher mouse [14]. Bone marrow transplantation (BMT) is one of the currently used treatments for the disease. It has been shown that BMT alone or in combination with gene therapy is associated with reduced CNS inflammation [9], [10]. There is also an associated reduction in pro-inflammatory cytokines and chemokines that correlate with the efficacy of therapy [10], [15], [16].

We show here that Keratinocyte Chemoattractant factor (KC) is greatly elevated in the brain and spinal cord of Twitcher mice. We also showed previously that the levels of KC decrease in response to a relatively effective therapy [10]. Keratinocyte chemoattractant factor belongs to the CXC family of chemokines and is a potent macrophage and neutrophil chemoattractant [17]–[19]. Keratinocyte chemoattractant signaling through its receptor, CXCR2, synergizes with another oligodendrocyte mitogen, platelet derived growth factor (PDGF) to cause oligodendrocyte precursor cell (OPC) proliferation, but can also act independently to cause migration arrest [20]. Since oligodendrocyte death and dysfunction as well as inflammation are observed in the CNS of Twitcher mice, we investigated the role of KC and its receptor CXCR2 in the two inter-related aspects of the disease. Our experiments demonstrate that global deficiency of KC or CXCR2 does not significantly influence the progression of GLD in the Twitcher mouse. We also demonstrate that the lack of KC or the lack of signaling through the CXCR2 receptor on donor hematopoietic-derived cells does not affect the disease progression in a bone marrow transplant setting. Interestingly, we found that there are several other cytokines and growth factors that are upregulated in the Twitcher CNS (e.g. MIP-2, PDGF-BB and FGF-2). Since some of these growth factors and cytokines have redundant functions in CNS inflammation, demyelination, and remyelination, it is possible that they fully compensate for the lack of KC and CXCR2. This study defines the role of KC and CXCR2 in the progression of inflammation in the Twitcher mouse and highlights the redundancy inherent in the cytokine/chemokine system.

## Materials and Methods

### Animal Procedures

Heterozygous (GALC +/−) mice and mice expressing GFP under the control of the pCAGGS promoter (GFP mice) were obtained from The Jackson Laboratory (Bar Harbor, ME) and maintained under the supervision of M.S.S. at Washington University School of Medicine. Heterozygous CXCR2 mice (CXCR2+/−) on the C57Bl/6J background were a kind gift from Dr. Ann Richmond (Vanderbilt University, Nashville, TN). Heterozygous KC mice (KC+/−) on the C57Bl/6J background were a kind gift from Dr. Sergio Lira (Mount Sinai School of Medicine, New York, NY). All the mice used in this study (KC−/−, CXCR2−/−, GALC−/− and GFP transgenic mice) were congenic on the C57Bl/6J background.

The mice were housed under standard conditions in a pathogen-free facility with *ad libitum* access to food and water. The Twitcher mice (GALC−/−) were obtained by heterozygous matings between GALC+/− mice. The breeding strategy that was used in order to generate the other experimental animals was as follows: Homozygous KC−/− mice were bred with heterozygous Twitcher (GALC+/−) mice. Progeny were screened for double heterozygotes (KC+/−GALC+/−). The double heterozygote mice (KC+/−GALC+/−) mice were bred to obtain KC−/−GALC+/− mice. Experimental animals (KC−/−GALC−/−) were generated by crossing the KC−/− GALC+/− mice with mice of the same genotype. Within the same colony, KC+/+GALC+/− animals were used as breeders to obtain KC+/+GALC−/− animals. In order to obtain CXCR2−/−GALC−/− mice, a similar strategy was used, except that the experimental animals were generated by breeding CXCR2+/−GALC+/− male and female, or CXCR2+/−GALC+/− female with CXCR2−/−GALC+/− male. The CXCR2-deficient mice were maintained on antibiotic water (Trimethoprim/Sulphamethoxazole) as they are known to be susceptible to infections.

To generate Twitcher-GFP mice, GFP transgenic mice (GFP+) were bred with GALC+/− mice. The bone marrow donors were generated by crossing GALC+/− GFP+ or GALC+/+GFP+ mice. To generate CXCR2−/−GFP+ mice, the CXCR2+/− mice were bred with GFP+ mice. The CXCR2+/−GFP+ mice were bred to generate CXCR2−/−GFP+ mice. These mice were maintained as separate colonies and were used as donors for bone marrow transplantation.

### Genotyping

PCR for GALC was done using the protocol described previously [21]. The following primers were used for genotyping KC [17]: 5′- GAA GAC AGA CTG CTC TGA TGG CAC -3′ and 5′-CCC TTC TAC TAG CAC AGT GGT TGA-3′. The following primers were used for genotyping CXCR2 [18]: 5′-CCT CGT ACT GCG TAT CCT GCC TCA G-3′ and 5′-TAG CCA TGA TCT TGA GAA GTC CAT G-3′. The lack of KC or CXCR2 was confirmed by the presence of NeoR cassette in the same PCR reaction. The NeoR primers used were: 5′-GGA TTG CAC GCA GGT TCT-3′ and 5′-GGA CAG GTC GGT CTT GAC AAA-3′. Homozygous deletion of KC protein was confirmed in two PCR-identified founder mice through Bio-plex kit (Bio-Rad laboratories, Hercules, CA). Homozygous deletion of the CXCR2 receptor was confirmed by flow cytometry of bone marrow from two PCR-identified CXCR2−/− mice using anti-mouse CXCR2-APC antibody (R & D systems, Minneapolis, MN). GFP phenotype was determined using an ultraviolet lamp held on the ventral surface of the newborn mice to detect fluorescence under the skin.

### Bone Marrow Transplantation

Animals were genotyped by PCR on postnatal day 9 using previously published protocols [17], [18], [21]. Nine-day-old mice received 900 rads of total body γ-radiation from a ^137^Cs source for conditioning followed by intraperitoneal injection of 3–4×10^7^ GFP(+), unfractionated bone marrow cells approximately 24 hours after irradiation [22]. Post-transplantation antibiotics included trimethoprim/sulfamethoxazole added to the water. Bone marrow donors were sex-matched GALC+/+CXCR2+/+, GALC +/+CXCR2−/−, or GALC−/−CXCR2+/+ mice, all expressing GFP under the CAGGS promoter [23].

### Flow Cytometry

Flow cytometry was used to quantify the hematopoietic-derived cells in the CNS and measure bone-marrow chimerism with donor-derived GFP+ cells after transplantation. For quantifying the hematopoietic-derived cells in the CNS, perfused mice brains were homogenized with collagenase/DNase buffer and passed through a 70 µm strainer [24], [25]. Collagenase/DNase buffer was made using 50 mg/ml collagenase D stock, 100 ug/ml TLCK trypsin inhibitor stock (Sigma, St. Louis, MO), DNase I 1 mg/ml stock, 1 M Hepes, pH 7.4 and Hank’s buffered salt solution. Collagenase D stock was made by dissolving 100 mg collagenase in 2 ml of TESCA buffer (50 mM TES, 0.36 mM Calcium chloride, pH7.4 at 37°C). DNase stock was made by dissolving bovine pancreatic DNAase (Sigma, St. Louis, MO) in 0.15 M NaCl.

The hematopoietic-derived cells were isolated from the homogenate by separation on a percoll gradient. Total number of cells isolated were estimated by sampling 20 µl of cells and counting them using a hemocytometer. The cells were stained with fluorophore-conjugated antibodies after Fc receptor block (BD biosciences, San Jose, CA). The following cells were identified and quantified by flow cytometry: activated microglia/macrophages (CD45^hi^ CD11b+), resting microglia (CD45^lo^ CD11b+), CD8+ T-cells, CD4+ T-cells [26], [27] and Neutrophils (Gr1^hi^ F4/80–) [28]. The data was acquired on a FACSCalibur flow cytometer (BD biosciences, San Jose, CA) using Cell Quest software (BD biosciences, San Jose, CA) and analyzed using FloJo software (Tree Star, Inc., Ashland, OR). Individual cell counts were obtained by multiplying the percentages of the various cell populations obtained by flow cytometry with the total cell counts. A total number of 3–6 mice per group were used for analysis. Spleen and bone marrow cells were used for positive controls. For quantifying donor engraftment, bone marrow was harvested from the femur and the percentage of GFP+ cells was determined.

### Cytokine Sandwich Immunoassays

The methods used in this study are as described previously [10], [29]. Briefly, animals were perfused with ice cold PBS after deep anesthesia. The brains and spinal cords were collected and homogenized in 10 mM Tris, 150 mM NaCl, 1 mM Dithiothreitol, 0.2% Triton-X100 and 20 µl/ml of Protease Inhibitor Cocktail (P8340, Sigma, St. Louis, MO). The supernatant was diluted to 0.5–2 mg protein/ml and the samples were stored at −70^o^C until use. The total n of 3–4 samples were used per group. The concentration of various cytokines and chemokines was determined using Bio-plex kit (Bio-Rad laboratories, Hercules, CA). The 23-plex sample kit includes the standards and antibodies for the following cytokines: IL-1α, IL-1β, IL-2, IL-3, IL-4, IL-5, IL-6, IL-9, IL-10, IL-12 (p40), IL-12 (p70), IL-13, IL-17, Eotaxin, G-CSF, GM-CSF, IFN-γ, KC, MCP-1, MIP-1α, MIP-1β, RANTES and TNF-α. A 3-plex kit for analyzing MIP-2, FGF-2 and PDGF-BB was separately used. The supernatant from brain and spinal cord homogenates was incubated with the fluorescent beads, washed and then incubated with biotin-labeled antibody cocktail. The samples were then incubated with streptavidin-Phycoerythrin and the fluorescence values were read in the Bio-Plex 2200 system (Bio-Rad laboratories, Hercules, CA). Standard curves were generated for each cytokine using the standards supplied with the kit and the individual cytokine concentration in each sample was estimated. Protein concentrations of the samples were determined using the Coomasie dye-binding assay (Bio-Rad, Hercules, CA).

### Histology and Immunofluorescence

For oligodendrocyte proliferation studies, the animals were injected with 5 mg/kg BrdU (Bromodeoxyuridine; Sigma, St. Louis, MO) every 8 hours for four days starting on day 32 of age. The lumbar spinal cords were collected after perfusion of the animals with PBS and 4% paraformaldehyde. The tissue was fixed in Enhanced Decalcification Formulation (SL85-32, Statlab, Lewisville, TX) for 2 days and cryoprotected in 30% sucrose. The tissues were then frozen in O.C.T. compound (Sakura Finetek, Torrance, CA) and cryosectioned. For immunostaining, the sections were co-stained with 1∶50 dilution of a rabbit anti-NG2 antibody (ab5320, Millipore, Billerica, MA) and 1∶100 dilution of mouse anti-BrdU (B2531, Sigma, St. Louis, MO) overnight at 4^o^C. The primary antibodies were detected using an anti-rabbit antibody conjugated to Alexafluor 555 (A-21428, Invitrogen, Carlsbad, CA) and a goat anti-mouse antibody conjugated to Alexa 488 (A-11001, Invitrogen, Carlsbad, CA). The images were acquired using a Ziess laser confocal microscope (Carl Ziess Microimaging, LLC, Thornwood, NY). The images were acquired using LSM/Axioskop software (Carl Ziess Microimaging, LLC, Thornwood, NY). Ten sections from each group with n = 4 animals per group were used for analysis. The cell counts were done manually. For luxol-fast blue (LFB) and periodic acid-Schiff (PAS) staining, the tissues were fixed overnight in 4% paraformaldehyde after perfusion with ice cold PBS and then transferred to 30% sucrose. The tissues were embedded in paraffin and the LFB and PAS staining was done using standard methods.

### Statistical Methods

GraphPad prism (GraphPad Software, Inc., La Jolla, CA) was used for statistical analyses and for generating graphs. Two-way unmatched ANOVA followed by post-hoc Bonferroni comparisons were used for analyzing the cytokine/chemokine data. One-way ANOVA followed by post-hoc Bonferroni comparisons were used for comparing various groups analyzed by FACS and for the analysis of oligodendrocyte progenitors. Log-rank test was used to compare the Kaplan-Meier survival curves. For statistical analysis of body weights, repeated measures ANOVA could not be used because of attrition, therefore one-way ANOVA at pre-determined time points was used instead.

## Results

### Altered Cytokine Profiles

**Figure 1:**
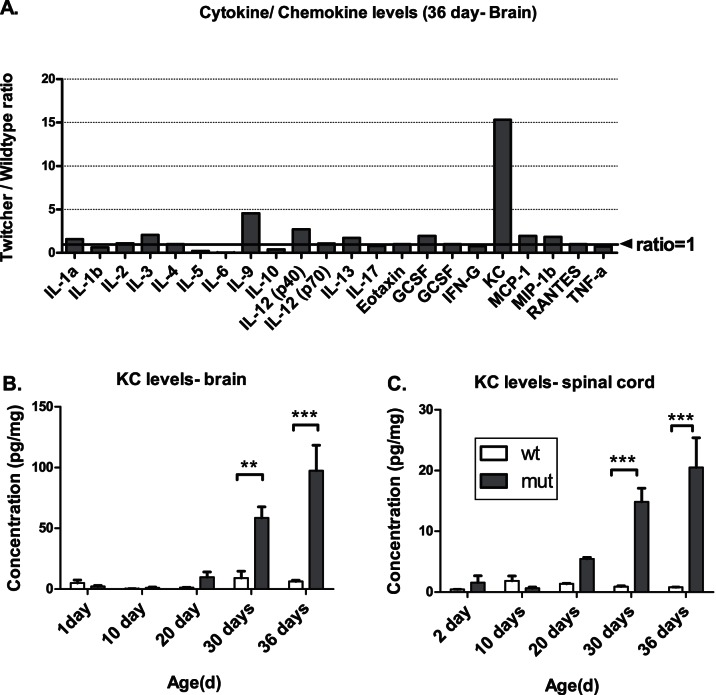
Cytokine and chemokine levels in the CNS of Twitcher mice. The fold-elevation of various cytokines/chemokines in the brain is shown. Among all the assayed molecules, the chemokine KC showed the greatest fold change in the brains (>15-fold increase) of the Twitcher mice (A). The levels of KC in the brains (B) and the spinal cords (C) of the Twitcher mice showed a progressive increase with time. The vertical bars represent the means and the error bars represent one SEM; **p<0.01, ***p<0.001.

Since inflammation is a prominent feature of GLD, and previous studies [10], [12], [30] have shown alterations in certain cytokines, a more comprehensive survey of cytokines and chemokines in the brains and spinal cords of Twitcher mice was performed at various time points. Several cytokines/chemokines were altered in the brains and spinal cords of Twitcher mice at different time points (Figure 1). Among the altered molecules, the chemokine KC was elevated 16–25-fold in the Twitcher brain and spinal cord compared to the wildtype (Figure 1). This elevation is progressive in both the brains and the spinal cords of Twitcher mice (Figures 1B and C). Other cytokines that were altered significantly include IL-12 (p40) and IL-9 in the brain (Figure 1A). Several other, non-statistically significant changes were observed, including an increase in the cytokines IL-3, G-CSF, MCP-1, and MIP-1B and a decrease in the cytokines IL-5, IL-6 and IL-10 in the brain (Figure 1A).

### Inflammation, Histology and Lifespan in KC−/−GALC−/− Mice

**Figure 2:**
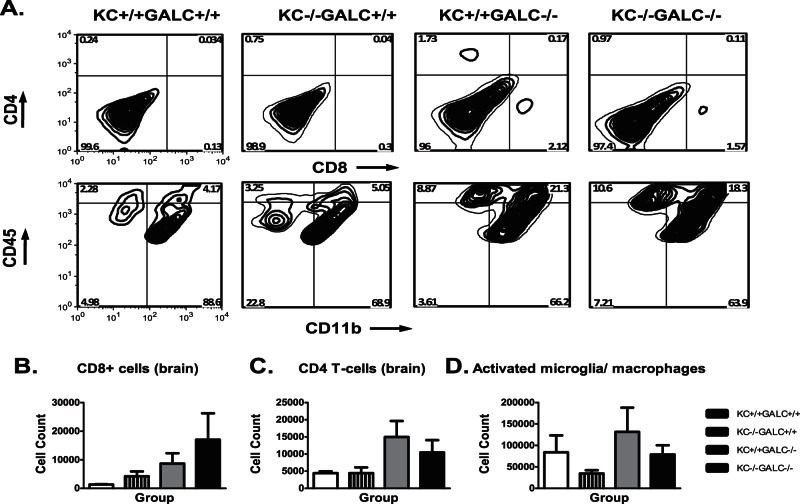
Flow cytometric characterization of inflammatory cells in the KC−/−GALC−/− brains. The upper row of Panel A contains representative bivariate contour plots showing CD8 and CD4 T-cells at day 36 in various groups of mice. The lower row in panel A contain representative bivariate contour plots showing activated microglia (CD45^hi^ CD11b+, upper right quadrant) isolated from the brain at 36 days of age. There is no significant increase in CD8+ T-cells in KC−/−GALC+/+, KC+/+GALC−/− or KC−/−GALC−/− mice compared to the KC+/+GALC+/+ mice (B). There is no significant difference in the CD4+ T-cells in the brains of the KC−/−GALC−/− and KC+/+GALC−/− mice compared to the KC+/+GALC+/+ mice (C). There is no significant difference between the KC+/+GALC−/− mice and KC−/−GALC−/− mice in the number of activated microglia (D).

**Figure 3:**
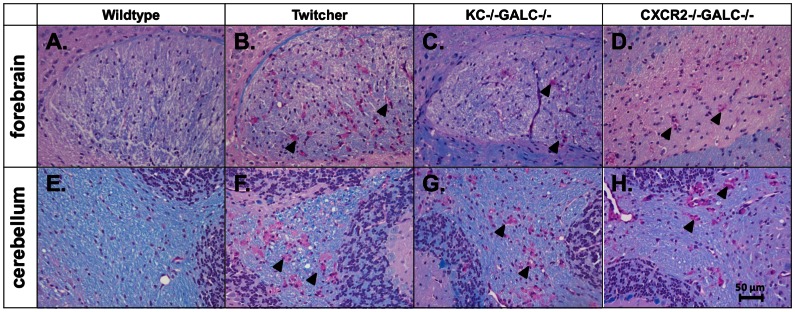
LFB/PAS staining of Twitcher mice lacking KC or CXCR2. Histology of the brains showing LFB staining (blue) and PAS staining (pink) in the corpus callosum (A-D) and cerebellum (E-H) in the wildtype, Twitcher, KC−/−GALC−/− mice and CXCR2−/−GALC−/− mice. The KC−/−GALC−/− forebrain (C) and cerebellum (G) show histology which is essentially identical to the Twitcher mice (KC+/+GALC−/−; B and F) with similar myelin staining and distribution of globoid cells. The CXCR2−/−GALC−/− mouse brains also demonstrate similar histology (D, H) compared to the Twitcher mice (B, F). There is no apparent difference between the KC+/+GALC+/+ and KC−/−GALC+/+ or the CXCR2−/−GALC+/+ mice in all the sections examined (images not shown). Scale bar equals 50 µm.

Given the role of KC in macrophage chemotaxis and oligodendrocyte development and the dramatic elevation in the Twitcher mouse brain and spinal cord, we hypothesized that KC contributes to the pathogenesis of GLD. In order to test this hypothesis, we generated mice lacking KC and GALC (KC−/−GALC−/−). Since elevated KC correlated with an increase in activated microglia/macrophages (CD45^hi^CD11b+) in the Twitcher mice [10], we further hypothesized that Twitcher mice lacking KC would have decreased activated microglia/macrophages in the CNS and a milder disease course. Surprisingly, KC−/−GALC−/− mice did not show any quantitative differences in the various inflammatory cells in the brain or spinal cord, when compared with that of KC+/+GALC−/− mice (Figure 2). The inflammatory cells from the brains and the spinal cords of the various groups of mice were isolated and stained for CD4, CD8, CD11b and CD45 using flourophore-conjugated antibodies. These cells were quantified using flow cytometry (as described in Materials and Methods). The upper row in Figure 2A shows the contour plots of CD4 and CD8 T-cells in the brain. The upper left and the lower right quadrants in each of these bivariate plots represent CD4 and CD8 T-cells respectively. Similarly, the lower row in Figure 2A shows the bivariate contour plots for cells stained with CD11b and CD45 flourophore-conjugated antibodies. The lower right and the upper right quadrants represent resting microglia (CD45^lo^CD11b+) and activated microglia (CD45^hi^CD11b+) respectively. There is no significant difference between KC−/−GALC+/+ and the KC−/−GALC−/− groups in terms of the CD4, CD8 and the activated microglial numbers. Similar results are seen in the spinal cords, except for a significant increase in CD4 T-cells in the spinal cords of KC+/+GALC−/− mice compared to KC−/−GALC−/− mice (data not shown). In addition, histology of the brain and spinal cord did not reveal any major differences when examined using LFB (myelin) or PAS (globoid cells) staining (Figure 3C and G). Finally, there was no alteration in the lifespan or body weight of KC−/−GALC−/− mice when compared with the KC+/+GALC−/− mice (data not shown).

### Inflammation, Histology and Lifespan in CXCR2−/−GALC−/− Mice

The similarity in the inflammatory profile between Twitcher mice with and without KC could be due to redundancy amongst the receptors for KC. KC is known to bind two chemokine receptors, CXCR1 and CXCR2 [31]. The receptor CXCR2 is more widely distributed throughout the CNS compared to CXCR1 and multiple ligands act on the CXCR2 receptor [32]. Deletion of CXCR2 will eliminate signaling by KC through that receptor as well as signaling by other molecules that could compensate for the action of KC. Therefore, we would expect that the consequences of CXCR2 deletion might be more dramatic than CXCR1 deletion. We hypothesized that Twitcher mice lacking CXCR2 would have decreased activated microglia/macrophages in the CNS and a milder disease course. Therefore, we generated CXCR2−/−GALC−/− mice in order to test the above hypothesis. When the brains of CXCR2−/−GALC−/− mice were compared to CXCR2+/+GALC−/− mice, there was no qualitative difference in LFB/PAS staining between the two groups (Figure 3D and H). There was also no difference between the CXCR2-deficient Twitcher mice and CXCR2-positve Twitcher mice in the profile of inflammatory cells in the brain as measured by flow cytometry (data not shown). Finally, there was no difference in lifespan or body weight in the CXCR2−/−GALC−/− mice when compared to CXCR2+/+GALC−/− mice (data not shown).

### Role of KC and CXCR2 in Oligodendrocyte Proliferation

**Figure 4:**
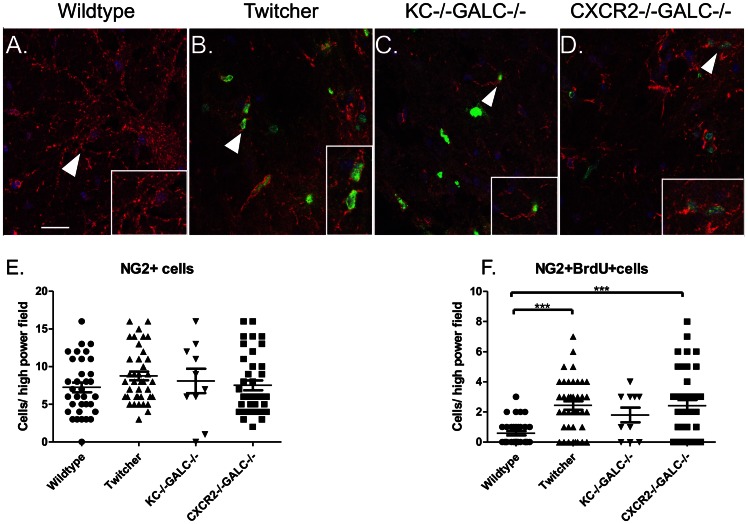
Oligodendrocyte precursor proliferation in the lumbar spinal cord of twitcher mice lacking KC or CXCR2. Arrowheads in panels A-D identify oligodendrocyte precursors expressing NG2 (red) with a more dendritic morphology. Arrowheads also identify the regions highlighted in the higher magnification insets. Minimal BrdU staining (green) was noted in wildtype mice (A) (scale bar, 25 µm). In B, C and D, the arrowheads identify proliferating oligodendrocytes that co-stain for NG2 (red) and BrdU (green). Blue represents nuclear staining with DAPI. There is no statistically significant difference in the total number of NG2+ cells in the spinal cords of various groups (E). A statistically significant (*** p<0.001) increase in number of NG2+BrdU+ cells/field is seen in the Twitcher and CXCR2−/− GALC−/− spinal cords compared to WT mice (F). There is no significant difference in the number of NG2+BrdU+ cells in KC−/−GALC−/− mice compared to Wildtype, Twitcher, or CXCR2−/− GALC−/− groups.

The striking elevation of KC in the brains and spinal cords of the Twitcher mice seem to have no apparent effect on the cellular inflammatory profile in the CNS. Another important function of KC and CXCR2 is to promote the differentiation and proliferation of oligodendrocytes [20]. KC and CXCR2 have been shown to be involved in oligodendrocyte precursor proliferation and migration in other mouse models of demyelinating diseases such as the cuprizone model, Jimpy mice (defective proteolipid protein), and mice with Theiler’s virus-induced demyelinating disease [33], [34]. Consistent with an increase in KC in the CNS, it has been reported previously that there is an increase in proliferating oligodendrocytes in the spinal cord of the Twitcher mice [35], possibly to replace those cells lost in the course of disease. Therefore, we hypothesized that KC elevation is important in promoting oligodendrocyte proliferation seen in the Twitcher spinal cord. We quantified the number of oligodendrocyte precursors (NG2+ cells) and the number of proliferating oligodendrocyte cells by Brdu/NG2 double immunostaining (Figure 4). There is no significant difference in the total number of NG2+ cells in the spinal cord of wildtype, Twitcher, KC−/−GALC−/− or CXCR2−/−GALC−/− mice (Figure 4E). However, we found a significant increase in the number of NG2+BrdU+ cells in the Twitcher spinal cord, compared to wildtype (Figure 4F). There was no significant difference in the number of NG2+BrdU+ cells among any of the GALC−/− groups (Figure 4F). This suggests that the lack of KC and CXCR2 does not significantly influence the proliferation of the NG2+ oligodendrocytic cells in the spinal cord.

### Selective CXCR2 or KC Deficiency in the Bone Marrow or CNS of Twitcher Mice does not Alter the Disease Course

**Figure 5:**
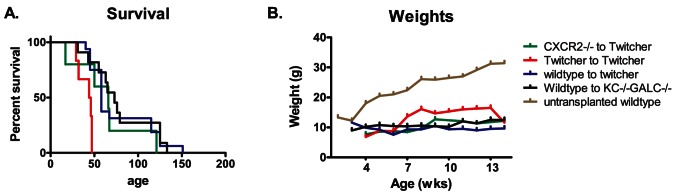
Effect of CXCR2 and KC bone marrow chimeras on the progression of GLD. The survival of Twitcher mice transplanted with CXCR2-deficient bone marrow (green, GALC+/+CXCR2−/− to Twitcher) or KC−/−GALC−/− mice transplanted with wild type bone marrow (black, wildtype to KC−/−GALC−/−) is not significantly different from Twitcher mice receiving wild type bone marrow (blue, wildtype to twitcher) (A). Twitcher mice transplanted with Twitcher bone marrow (red, Twitcher to Twitcher) have a similar survival (median age = 45 days) compared to untransplanted Twitcher mice (data not shown). The weights of various bone marrow chimeras are not significantly different from each other, but are significantly decreased compared to the wild type controls (B).

Previous studies have shown that CXCR2+ cells are involved in demyelination. Transplantation of CXCR2-deficient bone marrow decreases the severity of demyelination in the cuprizone model [36], [37]. In the current study, global lack of KC or CXCR2 in Twitcher mice does not alter the activated microglia/macrophages or prevent demyelination. However, it is possible that the beneficial effect of KC and CXCR2 deficiency in the bone marrow and other peripheral tissues are negated by their lack in the CNS or vice-versa, where they may be important in promoting repair. Therefore, we hypothesized that selective deficiency of CXCR2 in the bone marrow compartment or global deficiency of KC, including the CNS, would lead to decrease inflammation and alter the course of the disease. In the Twitcher mice, bone marrow transplantation supplies GALC activity to the CNS, and by itself prolongs the lifespan [38], [39]. Therefore, appropriate transplantation groups were used to control for this therapeutic effect. Twitcher mice transplanted with GALC+/+ CXCR2−/− bone marrow did not have an increased lifespan compared to Twitcher mice transplanted with GALC+/+CXCR2+/+ bone marrow (Figure 5A). Likewise, GALC−/−KC−/− mice transplanted with GALC+/+CXCR2+/+ bone marrow did not have a significantly increased lifespan. Hematopoietic chimerism determined at 36 days of age (26d post-transplant), showed that the engraftment of various groups is between 40 and 60% (data not shown). There was also no difference in the weights of the various groups of Twitcher mice that received transplantation (Figure 5B).

### Elevation of other Chemokines and Growth Factors

**Figure 6:**
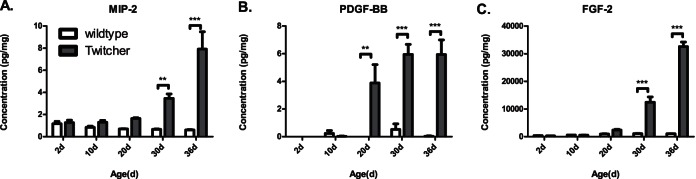
Altered cytokine and growth factor levels in the spinal cord of Twitcher mice that could possibly compensate for the lack of KC or CXCR2. MIP-2 (CXCL2) (A), PDGF-BB (B), and FGF-2 (C) levels are significantly and progressively elevated in the spinal cords of the Twitcher mice. Vertical bars represent the mean and the error bars represent one SEM (**p<0.01, ***p<0.001).

Since neither KC nor CXCR2 deficiency in the CNS had any measurable effects on the disease progression, other cytokines or growth factors, or both might be compensating for these deficiencies. Measurement of cytokines that could potentially act on CXCR2 (for e.g., CXCL2 or MIP-2), and other oligodendrocyte mitogens such as FGF-2 and PDGF-BB (Figure 6) show that there is a significant and progressive elevation in their levels with time in the spinal cord of Twitcher mice.

## Discussion

We show here that KC is highly elevated in both the brain and spinal cord of Twitcher mice. The elevation of KC in the Twitcher mouse was similar to that seen in other mouse models of demyelinating disease with infiltrating immune cells like Jimpy mice [34], Theiler’s encephalitis and EAE mice [40]. We previously showed that KC levels were nearly normalized in the brains of Twitcher mice following a relatively efficacious therapy (BMT combined with AAV-mediated gene therapy) [10]. A similar increase in KC was observed in an independent study performed in the Twitcher mouse [16]. In that study KC was not significantly decreased when Twitcher mice were transplanted with GALC+/+ mesenchymal stem cells (MSC). The differences between those two studies were the mode of treatment and the degree of response. The combination of BMT and gene therapy resulted in an increase in median life span of ∼82 days whereas the increase in life span following MSC transplantation was ∼5 days. Collectively, these data indicate that KC is responsive to a relatively effective treatment regimen and imply that KC might be involved in the disease pathogenesis.

Macrophage (globoid cell) infiltration into the brain and spinal cord is a hallmark of GLD, and it is believed to be pathogenic. Since KC and its receptor are known to be involved in macrophage recruitment [19], we hypothesized that increased levels of KC exacerbated the disease by recruitment and activation of microglia/macrophages into the CNS. In contrast to our prediction, there was neither a decrease in the number of activated microglia nor was there an alteration in the overall course of the disease in Twitcher mice lacking KC. Contrary to our expectation, there were activated microglia/macrophages in the CNS of the Twitcher mice lacking KC. This implies that KC is not required for the recruitment/activation of the microglia and macrophages or there are redundant pathways that can compensate for KC.

Keratinocyte chemoattractant factor functions through either the CXCR1 or CXCR2 receptor. Although, both CXCR1 and CXCR2 are known receptors for KC, only CXCR2 is expressed in the CNS [41]. Since the CXCR1 receptor is not as widely expressed in the CNS as CXCR2, we believed that CXCR1 would play less of a role in GLD compared to CXCR2. Furthermore, several other chemokines (CXCL1–3, 6 and 7) also act on CXCR2 [42]. It is possible that elevation of any of the other four ligands could compensate for the lack of KC, explaining the lack of effect observed by eliminating KC. Since there is considerable redundancy among the cytokines and chemokines, we hypothesized that any compensatory effects of other ligands binding to CXCR2 could be determined by studying the progression of disease in Twitcher mice lacking the chemokine receptor CXCR2. Similar to KC-deficiency, there was no alteration in the globoid-cells or the overall course of the disease in the CXCR2−/−GALC−/− mice compared to the CXCR2+/+GALC−/− mice. This observation could be explained again by the redundancy in the chemokine system. It is possible that KC could act on its alternative receptor CXCR1 [41] and bring about the same effects in the absence of CXCR2. Unfortunately, the severity of the Twitcher and CXCR2-deficient phenotypes precluded the generation of a mouse triply deficient in GALC, CXCR2, and KC (or CXCR1).

The chemokine KC and its receptor CXCR2 are also known to be involved in oligodendrocyte proliferation. The role of KC and CXCR2 in other animal models of remyelination are complex [43]. The findings in this study are consistent with a previous study [35] showing that proliferating NG2+ oligodendrocyte precursors were increased in the spinal cords of Twitcher mice. However, in the current study, the lack of KC or CXCR2 had no effect on the number of proliferating oligodendrocyte precursors compared to Twitcher mice. Other growth factors like FGF-2 and PDGF-BB are known to affect oligodendrocyte differentiation and migration and may compensate for the lack of KC. Interestingly, both FGF-2 and PDGF-BB are highly elevated in the spinal cords of Twitcher mice.

In a recent study, it was shown that hematopoietic chimeras using CXCR2-deficient donor bone marrow have reduced demyelination in response to cuprizone exposure [37]. It appears that the lack of CXCR2 decreases the number of neutrophils (and possibly macrophages) that migrate into the CNS and subsequently reduces demyelination. When similar bone marrow chimeras were made in the GALC-deficient mice, no such effect was seen, possibly implying that the myelin damage in the Twitcher mouse is very profound with minimal to no effect of the immune system on the disease progression. Alternatively, the partial bone marrow chimerism obtained by irradiating 9–10 day old mice could spare enough CXCR2-positive cells in the bone marrow to cause demyelination.

To summarize, although KC is highly elevated in the CNS of the Twitcher mice, its deficiency, and the deficiency of its receptor CXCR2, has no apparent effect on the inflammation, oligodendrocyte proliferation, or on the overall progression of the disease. These findings highlight the profound and rapid nature of the disease in the Twitcher mice and emphasize the redundancy within the chemokine system. It appears that therapies targeting individual cytokine systems or oligodendrocyte proliferation/myelin repair would be ineffective if the primary enzyme deficiency is not corrected. Although effective therapies like BMT plus CNS-directed gene therapy tend to normalize KC levels, there may be additional and independent mechanisms by which the therapeutic effect occurs. The roles of these additional independent mechanisms have to be explored in future studies. A more complete understanding of these mechanisms would provide a better understanding of how therapies like BMT contribute to the therapeutic benefit in GLD.
